# The family caregiving; A Rogerian concept analysis of Muslim perspective & Islamic sources

**DOI:** 10.1016/j.heliyon.2024.e25415

**Published:** 2024-01-28

**Authors:** Martyarini Budi Setyawati, A.P John Parsons, Bobbi Laing, Andrew Lynch, Imam Labib Habiburahman, Farah Nuril Izza

**Affiliations:** a School of Nursing, Faculty of Health Sciences, Harapan Bangsa University, Banyumas, Central Java, Indonesia; b School of Nursing, Faculty of Medical and Health Sciences, The University of Auckland, Auckland, New Zealand; c School of Population Health, Faculty of Medical and Health Sciences, The University of Auckland, Auckland, New Zealand; d Faculty of Islamic Law, Nahdlatul Ulama University Purwokerto, Central Java, Indonesia; e Faculty of Ushuluddin, Adab, and Humanities UIN Prof. K.H. Saifuddin Zuhri Purwokerto Central Java, Indonesia

**Keywords:** Concept analysis, Family, Caregiving, Islam, Muslim

## Abstract

Despite the numerous concepts of caregiving discussed in the literature, there is still no agreed definition and concept of family caregiving from the perspective of Islam. This study aims to comprehensively define family caregiving from Islamic religious and Muslim cultural perspectives. Rodger's evolutionary model was used to generate content by analyzing and redefining concepts. A thorough examination of the relevant literature using Scopus, PubMed, Medline, and CINAHL databases also trusted sources offered a total of 52 articles and 8 books to be reviewed. Our study reveals that family caregiving is viewed as God's gift as important as an essential religious and cultural obligation in Islam, where humans are expected to deliver care for their families although they are unprepared. This can be motivated by aspirations for respect, love, responsibility, and a desire to recompense parents, as well as the belief that by doing so they will be rewarded in the hereafter. The provision of family caregiving leads to positive consequences such as living with hope, gaining rewards and achievement, but at the same time, it also causes devastated life. This research contributes to a new discourse on family caregiving based on Islamic literature which helps in the comprehension of the practices of Muslim communities worldwide.

## 1 Introduction

The enthusiasm about family caregiving issues is growing as concern increases with the development of aging populations, chronic diseases, and disabilities [1]. Worldwide millions of family members dedicate their lives as caregivers, supporting their loved ones who are older, sick, and or disabled and who are likely to rely on family caregivers to look after their health and well-being. As an integral part of maintaining family relationships, they also provide home health and medical care, provide links to the health system or serve as surrogate decision-makers. Family caregivers are therefore essential in effective health management, especially long-term service, and support [2].

Efforts have been made to uncover the key concepts involved in family caregiving. The pivotal study on concepts of family caregiving was done by Swanson et al. (1997) which provided views on definitions, antecedents, and indicators from the viewpoint of the caregiver role. Later, Carbonneau, Caron, and Desroier (2010) highlighted the positive aspects of family caregiving which emerged in a family caregiver's daily life through enrichment experiences of caring for a person with dementia [3].

However, Crist et al. (2019), discuss it more as a caregiving tipping point, i.e., the role of caregiving augurs an abrupt, severe, and absolute change event involving either the caregiver and/or care recipient in the family with elderly [4]. A more recent systematic review (for family caregiving in patients with advanced liver disease) has proposed a conceptual model by focusing on the burden and factors associated with the family caregiver's quality of life [5]. Family caregiving is sometimes associated with the concept of filial duties, particularly in Asian cultures where younger generations perceive it as a way to reciprocate for the kindness and support they have received. Confucianism prioritises the importance of filial obligation as a guiding principle for children [6]. James Legge's translation of Confucian classics introduces the notion of a Christian deity into the concept of filial devotion, so substituting the earthly father figure with the divine Father in Heaven. This transition holds all human dads responsible to a Just and Loving God [7]. Nevertheless, this notion becomes dubious when the individuals requiring care are not the parents themselves.

In contrast for believers in Islam religion, caregiving is seen as a guide and a way of life. A chief tenet in Islamic guidance is about how to take care of the family, and this is rated as the second obligation of human obligations [8]. Providing good care to others can be interpreted as an example of devotion to the Islamic faith and beliefs. However, Muslim communities that share the Islamic religion have diverse cultures, ethnicities, and languages. Thus, the understanding and implementation of family caregiving will also vary according to the Muslim culture it is situated in. This diversity makes it challenging for nurses and healthcare providers to understand the most appropriate cultural (Muslim) practice which is true to Islamic principles and provides the best support for family caregivers.

Several scholars, from an Islamic perspective, have built on the concept of caring (rather than caregiving). For example, Barolia and Karmaliani (2008) involved an Islamic scholar's stance when they began exploring the concept of caring by proposing that caring can be seen as five interconnected and balanced elements: i.e., as physical, ethical/moral, ideological, spiritual, and intellectual dimensions of human personality. Some scholars also compared and contrasted the care perspective in Islam and Watson's concept of care [9].

Despite this developing body of knowledge, there is still no distinct definition and concept of family caregiving from the perspective of Islam. In the current literature, the perspective is taken from the point of view of nurses as the formal caregiver. Muslim families, therefore, require a clear religious interpretation of the concept of family caregiving as a paradigm to follow when they take care of their family members. Furthermore, this will help nurses, and healthcare providers discern their thoughts, motivation and practices of family caregiving. This study aims to clarify the foundations and family caregiving activities from Islamic religious and Muslim cultural sources and thus, provide a coherent and comprehensive definition of the concepts of family caregiving congruent with these.

## 2 Method

There are several ways to analyze and redefine concepts. One is Rodger's evolutionary model of concept analysis [10]. Rodger offers a conceptual analysis method that respects dynamism and attachment to reality. Through this analysis, the researcher will be led to higher levels of development and advancement for a term [10]. Given that the concept of family caregiving encompasses all of these dimensions, the conceptual analysis of Rodger is used here. This approach also offers the possibility to systematically examine concealed concepts [11]. The seven steps to follow in conducting this concept analysis are.

(a) Identify concepts of interest,
(b) Identify alternative surrogate terms and related uses of the concept,
(c) Choose the right data collection area,
(d) Determine the concept attributes,
(e) Determine references, antecedents, and consequences,
(f) Identify approach related to concepts interest,
(g) Model cases for identified concepts.

In general, Rodgers' approach uses thematic analysis as the direction for the study [12]. The Qualitative Data Analysis program NVivo by QRS International version 20 (QRS-NVivo 20) was utilized by the first and third authors to generate this content. Accordingly, all the articles were carefully examined, and pertinent phrases or words were gathered and organized under the headings “Attributes,” “Antecedents,” or “Consequences.” The acquired data were then analyzed repeatedly so that the authors could fully immerse themselves in the data and extract the crucial information and labels required to define each component of the in-question idea.

## 3 Results

### 3.1 Identifying concepts of interest

Family caregiving was chosen as a term. This is because in many of today's societies family caregiving has become more frequent and is becoming an essential, integral part along with advances in care management. This is a result of the increased number and life span of people with chronic disabilities or old elderly. This means defining the terminology surrounding caregiving is essential and worth discussing. Caregiving was referred to as a task, a transition, a position, or a process in a previous evaluation of the literature [13]. Moroney, 1998 has her own definition and says caregiving is a relationship-driven human service transaction between caregivers and care recipients. Through communication, resources are transferred and the emotional nurturing proces develops [14]. A definition taken from the Oxford Dictionaries states caregiving is “the activity or profession of regularly looking after a child or a sick, elderly, or disabled person” …... whereas family is “a basic social unit consisting of parents and their children, considered as a group, whether dwelling together or not” [15]. Hence, family caregiving is an activity of regularly looking after a child or a sick, elderly, or disabled family member (consisting of parents and their children, considered as a group, whether dwelling together or not). The caregiver in the context of family caregiving here is the father, mother, parents, siblings, or another kinship, while the care recipient is another family member who needs continuous care in a home setting [16]. Caregiving was also described by Hermanns and Mastel-Smith (2012) as adhering to the monens ponens logical argument (the way that affirms by affirming). A method of assisting someone who is unable to help themselves in a “holistic” (physically, cognitively, emotionally, and socially) manner is described as caregiving. It is enabled by specific characteristics, feelings, abilities, knowledge, time, and an emotional connection with the care recipient [17].

### 3.2 Identify alternative surrogate terms and related uses of the concept

Solving or interpreting the selected term will identify the surrogate term. The authors accomplished this by undertaking a preliminary literature review on the same topic, which focused on the experience of family caregivers. In order to discover articles about Muslim cultural perceptions, the authors employ several synonyms related to Islam, such as Islamic countries and those with a majority Muslim population, to broaden the pool of potential articles. Regarding Islamic religious sources, the authors selected the Quran and Al Hadith, which are used for spiritual guidance in Islam. Supporting literature, such as Tafseer or Interpretation of the Al Quran and Hadith by reputable Islamic priest, was also employed. The following words explain the surrogate terms used:

Family: Family, relatives, parent, sibling kinship, spouse, husband, wife, sister, and brother.

Caregiving: Caregiving, caring, care, treating, taking care, and looking after.

Islam religious and cultural perspectives: Muslim, Oman, Qatar, Kuwait, Iran OR Iraq OR UEA OR Saudi Arabia OR Saudi OR Indonesia, OR Pakistan.

### 3.3 Choose the right data collection area

To identify an appropriate realm for data collection, a systematic and in-depth review of the literature was conducted on notions indicating the use of the terms of this concept as applied in nursing and other healthcare databases [11]. Data collection was carried out from four databases: Scopus, Pubmed, Medline, and CINAHL (supplementary document 2). The literature search was not limited by the year of publication because the authors wanted a comprehensive and longitudinal point of view. The Islamic religious sources were picked from King Fahd University Library, (which contains English and Arabic languages) from textbooks or the e-catalog collection of Hadith*,* (the embodiment of the sunnah, the words and actions of Muhammad and his family) *shahih Muslim*. The search for articles and databases was based on terms and surrogate terms related to caregiving, as mentioned above. Text in English was set for the articles, while the English and Arabic versions of the Quran, Hadith and Tafsir were used.

**Fig. 1:**
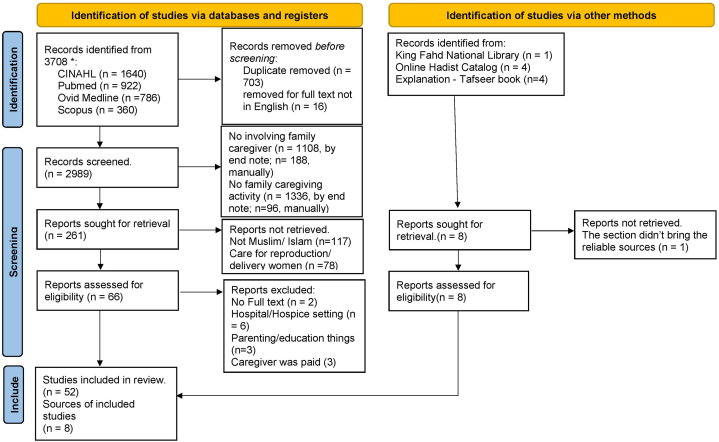
Flowchart of the assessment process.

A total of 3708 articles were found. Rigorous assessments were conducted by first, second and fourth authors using the SPIDER method to determine the inclusion criteria. SPIDER has proven to be a tool that makes formulating search terms easy, as it naturally fits the critical elements of the search query [18]. The articles focusing on Muslim families who look after their sick, and vulnerable family members, including caregiving activities in a home setting, and peer-reviewed journal articles written in English were included (supplementary document 1). After removing all duplicates and non-English text, 2989 articles were selected, and based on the title and abstract, 261 articles were left to be examined from the full text. This assessment process was carried out systematically adapting the PRISMA flow chart (Fig. 1). Articles were excluded if they were about: paid caregivers, parenting/educational issues, based in a hospital or hospice setting, or the care recipient was dying or had died. This left 52 articles (Table 1). Islamic religious sources were examined for conceptual attributes using surrogate terms to find verses of the Al Quran or relevant narrations from the Hadith or Tafseer. To ensure rigour, the fifth and six authors, who are also Islamic scholars, conducted a double check between the English translation and the Arabic version. A total of eight Islamic sources, including the Qur'an and Hadist, were included.

**Table 1:** Defining key terms of family caregiving as referenced in the included literature.

No	Author	Year	Country	Caregiver Characteristic			Care Reciever Problem	Key Term
				Kinship	Age	Sex		
1	Akbarbegloo & Sanaeefar	2022	Iran	Mother [4]; father [3]; sister [1]; brother [1]; Child [5]; Spouse [9]	mean: 43.5 sd 7.6	N/A	The person with Covid-19 treated at home	Providing basic needs and nursing care, Burden, Cause Financial Issues, Obedience toward Allah, Patient lesson, Opportunity to do good
2	Akbari et al.	2018	Iran	Father 7, Mother 4,Sister3, Brother 1, Spouse 5	20->60	Male 8, Female 12	person with mental illness	Cause negative Affect or emotion, involving entire family, extended family, and community, Affect social life, Frustrating
3	Al-Bahri et al.	2018	Oman	children: 84; spouse 43; siblings:34; parent 4, other kinship: 20	18-65, mean 34 SD 9	male: 113; female:72	patient with cancer	Providing basic and nursing care
4	Alijani Renani et al.	2014	Iran	8 mothers, 2 father and 9 patient	N/A	2 males, 8 females	children with asthma	Reward and achievement, Resulting in self-satisfaction, Helping, A Test and Punishment, Believe in Qadarullah
5	Alqahtani et al.	2016	Saudi Arabia	Parent	23–60	N/A	children with epilepsy	A punishment, Culture influence
6	Arabiat et al.	2013	Jordania	all mother	25->55	all female	Children with cancer	Be presense, Confining, Obligation, Belief in Qadarullah
7	Arian et al.	2017	Iran	N/A	25–53	N/A	Patient with cancer in terminal phase	A Test, Believe in Qadarullah
8	Aroogh et al.	2020	Iran	Wives: 2, Son: 3; Daugther: 3; Daugther in Law: 4	N/A	male: 3, female: 9	Patient in the End of Life phase	Be present, Confining, Obligation, Belief in Qadarullah
9	Arora et al.	2020	Norway	8 daughter, 2 daugther in law	23–40	all female	Elderly or sick family member	A Test, Believe in Qadarullah
10	Ashrafizadeh et al.	2021	Iran	9 daughters,1 son, 1 husband	48, ±26.12	2 male, 9 female	Patient with Alzheimer	Commitment, A religious duty, Care deliberately, Moral, human, and filial duty, Priority Duty, Providing basic needs and nursing care, Providing spiritual remedies, Sharing responsibility, Frustrating, inconvenience, Praying for them, Causing negative Affect or emotion
11	Baghcheghi et al.	2019	Iran	14 wifes	35–66	all female	Patients receiving hemodialysis	Sacrifice, Involves the whole family, extended family, and community, Culture expectations, Confining obligation
12	Crabtree	2007	UEA	15 parents	N/A	N/A	children with developmental disabilities	Responsibility
13	Croot	2012	UK	7 mothers, 3 fathers, 1 grandfather	N/A	4 males, 7 females	Children with disabilities	Obligation, A religious duty
14	Daher-Nashif et al.	2021	Qatar	Sharia scholar and family caregiver, kinship N/A	N/A	N/A	Patient with dementia	Burden, Cause negative feelings and emotions, A Gift, A Punishment, Believe in Qadarullah
15	Dalir et al.	2020	Iran	27 mothers, 8 fathers, 3 siblings, and 2 grandmothers	20–60	N/A	children with CHD	Reward and achievement, Resulting in self-satisfaction, Providing spiritual remedies, Care as best as they can, Involves whole family, extended family, and community
16	Darban et al.	2021	Iran	3 siblings, 5 spouses, 2 daughters, 1 son, 2 mothers, 2 other kinship	24–65	N/A	patient with Schizophrenia	Being rewarded afterward, Supporting patient's obligation toward Allah, Pray for them, Protect patient dignity, Responsibility, A religious duty, Involves whole family, extended family, and community, Role modeling, obedience toward Allah
17	Ebadi et al.	2021	Iran	Six were the patient's wife, 7 were offspring and 2 were the patient's parents.	42.16 ± 48 (27–78 years)	were females (60 %)	patients receiving hemodialysis	Providing spiritual remedies, Believe in Qadarullah
18	Ebrahimi et al.	2017	Iran	closed relatives, husbands, wives, and parents	26–54	14 females and 4 male	Patient with MS	Being rewarded afterward, Giving a positive aspect, A Huge Task, Religious Responsibility, Involves whole family, extended family and community, A Gift,
19	Etemadifar et al.	2015	Iran	N/A	20–50	women (76.20 %)	Patients With Heart Failure	Give a positive aspect, Resulting in self-satisfaction, Burden, Cause negative feelings and emotions,
20	Hamedanchi et al.	2016	Iran	all parent	60–72 years	five women and five men	Adult patients with intellectual disabilities	Resulting in self-satisfaction, Living with Hope, Keep moving on, Providing spiritual remedies, Alternative treatment, A religious duty, Believe in Qadarullah
21	Hasnain & Rana	2010	US	1 daughter	N/A	1 female	people with dementia	Give a positive aspect, Burden, Cause negative feelings and emotions, Providing spiritual remedies, Providing basic and nursing care, Involves whole family, extended family and community, Commitment,
22	Hashemi-Ghasemabadi et al.	2016	Iran	4 husbands, 8 daughters, 3 sons, 2 parents, 6 other kinship	20 to 69	women (69.6 %)	Person with Cancer	Give a positive aspect, Burden, Cause negative feelings and emotions, Commitment
23	Hosseini et al.	2021	Iran	9 daughters,2 sons, 3 wife	mean 54.57 ± 14.64	female: 71.4 %; male 28.6 %	The person with Alzheimer's Disease	A punishment, A religious duty, Burden, Cause family conflict, Cause negative Affect or emotion, Culture influence, Effect on social life, Frustrating, Fulfilling expectations, Making their life plan change, Causing Financial Issue
24	Houlihan	2015	Iran	2Mother, 28 Husband, 5 Sister,12Daughter, 4 Son/brother	38.8 (10.9)	male 22 (53 %); female 19 (47 %)	Women with cancer	Keep moving On, Cause their life plan changed, Isolated, Cause negative feelings and emotions, Shock and unprepared, Providing basic and nursing care, Obligation, Responsibility, A religious duty, Involves whole family, extended family and community, Hard, Commitment, A Task,
25	Khorsandi et al.	2020	Iran	11 Mother, 6 Father	N/A	11 female, 6 male	Children with CKD	Resulting in self-satisfaction, Patience lesson, Giving a positive aspect, Being rewarded afterward, Keep moving On, Causing their life plan to change, Providing spiritual remedies, Care as best as they can, Obligation, Involves whole family, extended family and community, Religious duty, Hard, Opportunity to do good, Believe in Qadarullah, Moral, human and filial motivation, Showing their love to mother,
26	Kolmar et al.	2022	US	all mother	N/A	all female	children with life-limiting conditions (LLC)	Give a positive aspect, Emerging spiritual growth, Cause their life plan to change, Cause negative feelings and emotions,
27	Leichtentritt et al.	2004	Israel	5 Wife, 3 Daughters, 4 Daughters-in-law, 6 Sons,	N/A	12 female, 6 male	people with cognitive decline	Keep moving On, Cause their life plan changed, Fatigue and Physical burnout, Cause negative feelings and emotions, Shock and unprepared, Providing spiritual remedies, Providing basic and nursing care, Hard, Involves whole family, extended family, and community
28	Mandani et al.	2018	Iran	7 mother, 2 father, 1 wife, 2 sister, 1 brother, 2 other kinship	42–73	5 men and 10 women	people with a chronic psychiatric disorder	Keep moving On, Providing spiritual remedies, A religious duty, A Gift, A Test, Believe in Qadarullah,
29	Marshall P	2010	US	all mother	mean 38.5 years	30 women	children with intellectual and developmental disabilities	Being rewarded afterward, Being a decision maker, A religious duty, Involves the whole family, extended family, and community, Supporting and sharing responsibility, Showing their love to their mother, A Compulsion
30	Meshkinyazd et al.	2020	Iran	N/A	The mean age of 38.7 ± 6.5	4 male, 6 female	people with borderline personality disorder	Tied up the bond, Fatigue and Physical burnout, Cause negative feelings and emotions, Obligation,
31	Moradi et al.	2022	Iran	5 Mothers, 1 Grand Mother, Other therapies	34–60	10 female - 2 male	children with a cochlear implant	Patience lesson, Negative social impact, Confining obligation, A Huge Task, Religious Responsibility, Involves whole family, extended family, and community, Sacrifice,
32	Mortazavi et al.	2015	Iran	6 sons, 2 daughters, 1 son-in-law, q wife, 1 sister, 1 brother, 2 elder people itself	40–78	8 male, 6 female	elderly	Fatique and Physical burnout, Frustating, Desire to die, Confining obligation,
33	Mousavi et al.	2021	Iran	7 mother, 1 son, 1 wife, 1 husband, 1 sister, 1 brother	36–67	3 male, 9 female	people with bipolar I	Care Deliberately,
34	Nahal et al.,	2017	Palestina	all mother	29–62	all female	Children With Spina Bifida	Cause their life plan changed, Causing losing job, Cause family conflict, Cause negative feelings and emotions, Frustrating, Cause financial issues, Risk themself, Hard
35	Navab et al.,	2012	Iran	5 daughters, 2 wives, 1 husband	25–67	1 male, 7 female	person with AD	Frustrating, Shock and unprepared, Providing basic and nursing care,
36	Navab et al.,	2013	Iran	5 daughters, 2 wives, and 1 husband	25–67	1 male, 7 female	person with AD	Being rewarded afterward, Living with Hope, Keep moving On, Fatigue and Physical burnout, Isolated, Causing negative feelings and emotions, Providing spiritual remedies, Providing basic and nursing care, Care as best as they can, Obligation, Priority duty, A religious duty, Involves whole family, extended family and community, Hard, A task, A Gift, Believing in Qadarullah
37	Nayeri et al.,	2021	Iran	17 mothers, 17 father	20s–40s	17 male, 17 female	child with CHD	Negative Social impact, Burden, Bring shame,
38	Nemati et al.,	2018	Iran	4 Mothers, 1 Father, 1 sister, 1 brother, 4 husbands, 10 sons/daughters	22–67	8 Males, 13 female	cancer patient	Sin Abolishment, Burden, Cause negative feeling and emotions, Hard, Punishment,
39	Nematollahi et al.,	2021	Iran	14 mothers, and 6 fathers	24–55	6 male, 14 female	Children with Phenylketonuria	Fatigue and Physical burnout, Negative social impact, Frustrating, Cause financial issues, Providing spiritual remedies, Sacrifice, Hard, A Test, Believe in Qadarullah
40	Noveiri et al.,	2021	Iran	all husband	mean 51, 42-70	20 male	Cancer women	Cause their life plan changed, Causing family conflict, Cause negative feelings and emotions, Fatigue and Physical burnout, Frustrating, Cause financial issues, Shock and unprepared, Fulfill the expectation, Obligation, Hard,
41	Perngmark et al.,	2022	Thailand	adult son and daughters,	34–50	four were female and one male	older Adult with some disease	Patience lessons, Giving a positive aspect, Being rewarded afterward, Providing spiritual remedies, Hard, A Gift, A Test, Believing in Qadarullah
42	Ragsdale et al.,	2018	US	6 fathers, 11 mothers, 1 grandmother	N/A	6 male, 12 female	Children with Bone Marrow Transplantation	Keep moving On, Cause their life plan changed, Causing negative feeling and emotions, Cause financial issues, Care as best as they can, Hard
43	Rahimi et al.,	2021	Iran	mother, father, child, sister, and spouse	22 to 54	1 male, 12 female	The person with Covid at home setting	A religious duty, Involves the whole family, extended family and community,
44	Saimaldaher & Wazqar,	2020	Saudi Arabia	59 spouses, 45 parents, 14 siblings, 42 children	mean 35.4	62 male, 98 female	Adult patient with cancer	Patience lesson, Being rewarded afterward, Providing spiritual remidies, Believe in Qadarullah, A Test, A Gift, Hard
45	Salehitali et al.,	2018	Iran	father, mother, daughter, son, spouse, or grandfather	25–70 years	7 males and 9 females	patients undergoing hemodialysis	Give a positive aspect, Emerging spiritual growth, Cause negative feelings and emotions, Cause financial issues, Shock and unprepared,
46	Salehi-Tali et al.,	2018	Iran	father, mother, daughter, son, spouse, or grandfather	25–70 years	7 males and 9 females	patients undergoing hemodialysis	Fatigue and Physical burnout, Cause financial issues, Confining,
47	Sharif et al.,	2020	Saudi Arabia	3 mothers, 1 father, 1 husband, 1 daughter, 1 son, 2 brothers, 3 sisters, 1 uncle	21–65	3 male, 10 female	people with mental disorders	Resulting in self-satisfaction, Patience lesson, Keep moving On, Providing basic and nursing care, Care as best as they can, Obligation, Protecting patient dignity, Culture expectation, Important, Commitment, Inherent Love, Hard
48	Taleghani et al.,	2012	Iran	4 Father, 11 Mother	25–45 years	4 male, 11 female	Children with cancer	Cause their life plan changed, Causing losing job, Cause family conflict, Fatigue, Physical burnout, Cause negative feelings and emotions, Cause financial issues, Providing basic and nursing care, Long journey, Hard,
49	Tavakol et al.,	2018	Iran	N/A	26–56 years	8 females and 4 males	Children with disability	Patience lessons, Give a positive aspect, Being rewarded afterward, Causing their life plan to change, Isolated, Fatigue and Physical burnout, Causing negative feelings and emotions, Providing spiritual remedies, Providing basic and nursing care, Obligation, A Gift, Hard, A Test, Believe in Qadarullah
50	Yoosefi lebni et al.,	2021	Iran	All mother	19 and 50 years	all female	Children with disabilities	Cause family conflict, Punishment
51	Yunita et al.,	2020	Indonesia	4 mother, 2 Father, 1 sister, 1 brother,2 aunties	37–65	2 males, 6 female	patients with mental disorders post- pasung	Cause family conflict, Punishment, Negative social impact, Fatigue and Physical burnout, Cause negative feelings and emotions, Bring shame, Cause financial issues, A Test, Believe in Qadarullah
52	Zahed et al.,	2019	Iran	1 friend, 4 wives, 9 daughters,1 son, 1 husband, 2 daughters-in-law, and 1nieces, or nephew	23 to 84	3 male, 19 female	people with dementia	Resulting in self-satisfaction, Causing their life plan to change, Cause losing opportunity, Negative Social impact, Cause family conflict, Religious duty, A Punishment

### 3.4 Determining attributes, antecedents and consequences

**Fig. 2:**
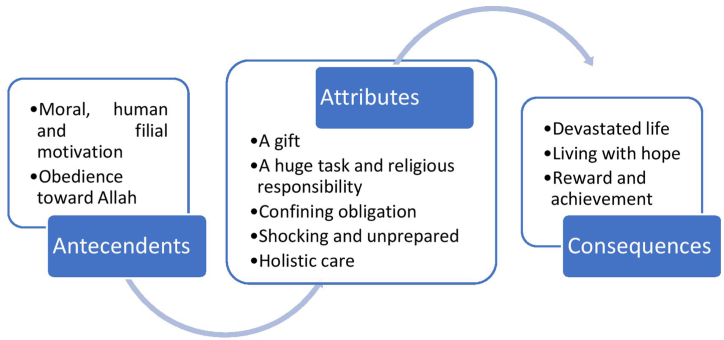
Conceptual model of defining attributes, Antecedents, and consequences of family caregiving based on Muslim.

After locating the data selection area and categorizing the articles and thematic analyses of all articles and literature (Table 2), the authors was able to provide an overview of the concept of family caregiving in Islam (Fig. 2).

**Table 2:** An example of defining attribute of the family caregiving.

Meaning units (excerpts from retrieved documents)	Definition	Sub-attributes	Defining attribute
“I say this is a divine test. I always say: God tests his servants with difficulties” (Khorsandi et al., 2020, p.6).	An examination of family caregiver’ s faith by doing activities for them to perform	A Test and Punishment	A gift
“Faith in Allah helped me through, it is Allah's gift… This is our destiny; we pray that God will reward us” (Nahal et al., 2017, p.241).	The situation that believes that it came from God	Allah Gift	
“I see this as a good opportunity for myself; an opportunity that I am very calm when I am with her and I am free from all worldly issues and works” (Hosseini et al., 2021, p.6).	The time when a particular situation makes it possible to do something valuable	Opportunity to do good	
“ Since I am a Sayyed (descendants of the prophet), it is my religious duty to be a caregiver for my family. I took care of her from the beginning until now and I feel satisfied. I vowed that when she recovers, I will bring her to Imam Hussein^a^ shrine in Karbala pilgrimage” (Ebrahimi et al., 2017, p.4).	A duty to take care of somebody so that the family caregiver may be blamed if something goes wrong and it came from their belief.	Religious Responsibility	A Huge Task and Religious Responsibility
“This is our way of showing our commitment to the values that were nurtured in our family” (Zahed et al., 2019, p.3).	Family caregiver feel or behave in a particular way as a promise to support patient (their own family)	A life time commitment	
“I see it as my duty” (Yoosefi lebni et al., 2021, p.1176).	Something that family caregivers feel they have to do.	A Duty	

a The grandson of Prophet Muhammad and Islamic leader.

#### 3.4.1 The attribute of family caregiving

The fourth step in concept analysis was to identify the characteristics of the concept that will lead to an actual definition. A review of the Islamic religious literature revealed that family caregiving was identified by several terms listed in the text below.

##### 3.4.1.1 A gift

Almost always, a gift is associated with anything that makes you joyful or enjoyable. Yet, according to Merriam-Webster's dictionary, gifts are defined as acts of gratuitous giving that may not be specified whether something is or not [19]. In the Islamic religious perspective, a gift of this nature is one granted by Allah upon his servant [20,21] which was narrated by participant “… taking care of the patient was a blessing in their life and a reward from God” [22]. The gift does not have to be anything enjoyable, but it can be something useful to the recipient as participant said “My faith, of course, tells you that whatever God gives you, It's not something you can't handle. and we don't know what the reason is” [21]. This is consistent with what is perceived by Alzheimer's family caregivers here [23]. In a broader sense, gifting can also be a perfect time to make amends for one's transgressions. A Muslim family caregiver of an individual with cognitive decline and disabilities feels that his misfortune is an opportunity for him to atone for his misdeeds [23,24]. On the other side, the opportunity is the available time to gather good deeds that would benefit him in the next life [25].

##### 3.4.1.2 A huge task and religious responsibility

Family caregiving is a difficult duty and a religious obligation, according to 30 of the 52 articles reviewed, making this the definition most frequently cited by Muslim family caregivers. As the male family caregiver stated, several of them noted that this responsibility is part of a lifetime commitment inside a marriage or out of respect for parents. “Maybe you won't believe it. I do not even let her do even a little bit of work at home because marriage means commitment and responsibility toward the person you are living with (Husband, 5 years of care) [26]. This concept is in 15 articles [[26], [27], [28], [29], [30], [31], [32], [33], [34], [35], [36], [37], [38], [39], [40]], the words hard were followed by the words duty, work, or responsibility, which implies family caregivers considered their responsibilities to be burdensome. In Mortazavi et al., 2015, participant stated “Finally, I called my brother living in another city and told him this is a reality, my wife has left me. My life is lost and I am responsible for my famiy and I can't go to work any longer. If I cannot work there would be no income …”. Another old person's son stated: “… well, we reached a deadlock and I stated that either my mother should be at our homes or I declare that I am at the end and …”. This exemplifies the difficult nature of their responsibilities as family caregivers, to the extent that it profoundly alters their life and engenders feelings of frustration. Moreover in one of these studies a participant selected the word “marar”, which means ‘life's bitterness’, to characterize family caregiving [32]. Yet, a number of other articles [33,[41], [42], [43]] identified family caregivers as “sacrificing” to their families. As stated by a Muslim family caregiver with a cardiovascular condition, the duty being performed by this individual was so burdensome that it could endanger his life, “I have heart disease, whenever I take him to the bathroom, I suffer some difficulties and my situation becomes worse” (31).

##### 3.4.1.3 Confining the obligation

Unquestionably, caring for sick family members increases the bond that is deep-rooted in their family role [28,29,[39], [40], [41], [42],[44], [45], [46], [47]]. It is derived from participant narratives:

“Caring for my father is an obligation on me, it is not a choice” [41].

“It is my duty to my father and I have to take care ofhim now” [29].

“Based on what I believed, we must give his medicine on time and take care of his diet, sleep, and situation” [46].

Some family caregiver also stated about the obligation was due to a force or situation as they said “The court obligated me to be my father's guardian when I was just 19” [39]. A Muslim woman stated clearly that this was ‘the chain’ that bound her, as the court had ruled that she was responsible for caring for her dying father [47]. She feels constrained not just by legal requirements but also by the gender norms imposed by the Muslim community in her neighbourhood. Similarly for whose family member suffers from a personality disorder as “a chain to the feet”(44). When they have to leave their sick family members for a longer time, they must repeatedly ensure that it is safe to do so [28,40,44].

The Al Quran explicitly delineates the duties and obligations of family members towards one another in 15 verses. These responsibilities are categorised into the responsibilities of men towards their wives in An Nisa:34 “Men are in charge of women by [right of] what Allah has given one over the other and what they spend [for maintenance] from their wealth. This means that a husband is responsible for his wife's welfare, including her well-being. Al Quran also mention about the obligation wives towards their husbands, and children towards their parents. The last point is the most frequently discussed one. As started in surah Al Ahqaf:15 “And We have enjoined upon man, to his parents, good treatment” or in Al Luqman:14 “We have enjoined upon man [care] for his parents. His mother carried him, [increasing her] in weakness upon weakness, and his weaning is in two years. Be grateful to Me and to your parents; to Me is the [final] destination” [53].

##### 3.4.1.4 Shocking and unprepared

Having the additional responsibility of caring for a family member was seen as one of the most unforeseen changes, at least in six studies [21,28,30,34,48,49]. The participants had a sense of unpreparedness in relation to their new roles. This emotion encompassed an unexpected recognition of a devastating ailment, perplexity over the caregiver's function, and the management of the circumstance and acquisition of novel obligations. According to them, the initial experience rendered them speechless, unsure of what to do or what to do next [28]. They only considered living the lives that had been imposed upon them. Other family caregivers laid the blame on the health system, which gave little guidance on how to prepare them to care for ailing family members at home [49]. Other Muslim family caregivers were still in the grieving phase, where they still experienced phases of anger, and denial [21]. This happens because participants are dealing with limited life conditions from their families so that the grieving phase is still present among the participants' narratives.

##### 3.4.1.5 Holistic care

Undoubtedly, Muslim family caregivers provided care for the body, mind, soul and assisted with nursing care at home as a part of their holistic care. They provided comprehensive assistance with activities of daily living, medication and treatment management, ensuring their safety and even their reputation in the community [28,49,50]. Notwithstanding the inconvenience, they continued to attend to the social requirements of the sick individual by bringing them to their friends and relatives [26].

Muslim family caregivers have a thorough understanding of the terms of caring. They acknowledged to doing all that was necessary to be able to provide the best care for their family members [26,32,51]. Several articles discovered that Muslim family caregivers also engaged in alternative therapy [25,37,51,52], specifically to release them from “Ain”, the demon disease (Ragsdale et al., 2018).

A spiritual treatment for the sick is prayer [25]. This is consistent with the Al -Quran “O you who have believed, seek help through patience and prayer. Indeed, Allah is with the patient ” (53). In addition, in the Hadist it is written, that one of the obligations of Muslims towards other Muslims is to pray for them [[54], [55], [56]]. In line with this, seven articles indicated that Muslim family caregivers do this to take care of the heart and spirituality of their sick relatives [21,25,35,37,50,57].

#### 3.4.2 Antecedents of family caregiving

##### 3.4.2.1 Moral, human and filial motivation

The intention behind caring for a sick family member is not questionable. Yet, suppose we are to have a better understanding of the caregivers' motivations for caring for a sick family member. In that case, it is appropriate to develop/modify interventions to protect the caregivers' altruistic drive and reduce the negative features of informal caregiving [40,58]. As articles clearly mention, these embody moral, human, and filial motivation, with filial motivation serving as the primary impetus for them to fulfil their tasks [29]. Fourteen of the articles addressed extended families or communities as family caregivers, with one article describing their moral and human motivations as Muslims who cared for people without blood connections. In Hadist stated ““The rights of the Muslim upon the Muslim are six, when you meet him, give him the greeting of peace, when he invites you, respond to his invitation, when he seeks your advice, advise him, when he sneezes and praises Allah, supplicate for mercy upon him, when he becomes ill, visit him and when he dies, follow him (i.e. his funeral)’ explain the moral responsibility toward humanities [56].

One article mention about being honour as a caregiver, as a participant stated: “My mother and I are taking care of a patient who has Alzheimer's disease because she is a descendant of the Prophet Mohammad (a Saadat) [40]. In Muslim culture, it is considered an honour to serve Prophet Mohammad's descendants. It probably would have been different if she was not a Saadat (families believed to be descendants of the Islamic prophet Muhammad). We serve this patient wholeheartedly [40].

Filial motivation is sometimes not based on sincerity, but rather obligation, such as when he is the only child living in the same area as his parents, and he has no choice but to take care of his elderly and chronically ill parents [39,59], especially when accompanied by economic conditions that make it impossible to hire a paid caregiver [59].

##### 3.4.2.2 Obedience toward allah

In the Qur'an, many verses regulate how those of Islamic faith have filial duty to their parents, or to their partners. For example in Al Isra 23, it is clearly stated that it is written that it is the duty of a child to be good to his parents:

“And your Lord has decreed that you not worship except Him, and to parents, good treatment. Whether one or both of them reach old age [while] with you, say not to them [so much as], "uff," and do not repel them but speak to them a noble word.” [53].

In one of the Hadiths, it states “Goodness towards (one's) parents is the greatest obligatory act”, to further clarify this. In this view, caring for parents and ensuring their well-being constitutes doing being good to them. In the study of Tawhid, obedience is emphasized, and Islam believers are required to be able to obey Allah when directed by Him [20]. Consequently, care for ill and vulnerable parents is evidence that Muslims are obedient to their God [20,21,25,60].

In addition, Muslims are obligated to believe in the presence of Al Qada and Al Qadar, where the scenario of a Muslim's life was predetermined before he was born, as stated in the Hadith. “Praise be to Allah that we are Muslims, and we know that everything is Qada'a [fate] and Qadar [destiny] and Allah has planned it all; everything is written with Allah. I mean that no man was born without his life being planned including when you're born, what afflicts you, and when you die” [37].

The family caregiver accepts the additional role given by God without complaint and lives it with trust, as said by one of the participants who cared for a family member with chronic illness [21,60]. Muslims are aware that everything is Qada'a [fate] and Qadar [destiny] and that Allah has planned it all; everything is written in Allah's book (i.e the Al Quran, Hadist). It means that no human was ever born without his/her life having been predetermined, including his birth, afflictions, and death. Muslim family caregivers accept whatever God, as the ultimate power, desires for their future. “They accept God's will and ask simply for patience” [60]. The family caregiver gives all to Allah without resistance or denial, as they remarked, " …. Accepting everything from God …. I have nothing to say; I have already given up” [52].

#### 3.4.3 Consequences of family caregiving

##### 3.4.3.1 Devastated life

Our lives will undoubtedly change when we take on new responsibilities or occupations. This happened to almost all Muslim family caregivers, who claimed they were forced to alter their life plans due to job loss [29,38], or miss out on other opportunities, such as the chance to pursue their dreams of attending college [28,29,38,39],. According to the son of older person with Alzheimer's disease, he had changed how he lives, works, and believes in the world [29].

As stated in the majority of articles, burden becomes integrated into the family caregivers role. This role puts a physical strain on family caregivers, who may become overwhelmed [61]. In fact, one of the participants said that he was no longer happy, considered leaving, and even hoped that he would eventually leave this world [38].

Family caregivers also deal with the social costs such as loneliness, isolation, and conflict with family members [23,24,33,42,62,63]. A participant claimed that because of the family caregiving role, his sister got divorced, because her husband forced her to choose between her mother and her husband [34]. An additional financial burden is created also because family caregivers can no longer provide proper medical treatment for their sick family members, as was stated, “We lost four hundred in a month for medication. After that, I sought a loan. There were no funds, so we were back home” [52].

##### 3.4.3.2 Living with hope

Although feeling a great deal of hardship, family caregivers continue to fulfil their responsibilities using the different resources and techniques they possess [21,26,[28], [29], [30],^32^,^36^,^60^]. For example, one participant who cared for Multiple Schlerosis daughter said “Many times I felt stress and exhaustion, but reminded myself to be hopeful for my daughter. I said to myself that I should be strong during difficulties and remain besides her.” He believed by focusing on hopefulness he can keep his resiience [60]. Another example came from family caregiver who attempted to address the issues with their father forgetting, with micro-management. When their father wanted to go to work, for instance, they would check to see if his phone was charged or if the office key was included [29]. One of the best coping strategies that a family caregiver can offer is to focus on the good and not let things that are not important bother them. This is also explained in the Qu'ran, about the importance of trying and not giving up. *“And they will say*, *“Praise to Allah*, *who has removed from us [all] sorrow*. *Indeed*, *our Lord is Forgiving and Appreciative”(53)*. This spiritual encouragement gives them strength to keep them moving on and living their lives optimistically [26].

##### 3.4.3.3 Reward and achievement

Prophet Muhammad said:

“Whoever removes a worldly grief from a believer, Allah will remove from him one of the griefs at the Day of Resurrection. And whoever relieves the need of a needy person, Allah will alleviate his needs in this world and the Hereafter. Whoever shields [or hides the misdeeds of] a Muslim, Allah will shield him in this world and the hereafter. And Allah will aid His slave so long as He aids His brother. And whoever follows a path to seek knowledge therein, Allah will make easy for him a path to Paradise …” [55,64].

There are great rewards for those who help others in need, either in this world or hereafter. Even though the assistance here is broad in nature, it is believed that family caregiving also includes helping people who are experiencing diseases or disabilities [53,56,65]. Family caregivers say that there are positive things to be gained from family caring for their sick family members [22,27,29,35,39,40,48,50,66,67]. They even think that through this family caregiving, they stimulate spiritual growth (Rahimi, [35,48,67].

Participants believe that what they will get a reward from Allah as stated by one of the participants “I will wait, and I will receive God's reward in the other world” [35], which is following The Qur'an sura al-Baqarah: 215 states:

They ask you, [O Muhammad], what they should spend. Say, “Whatever you spend of good is [to be] for parents and relatives and orphans and the needy and the traveller. And whatever you do of good - indeed, Allah is knowing of it [53].

### 3.5 Identify concept related to the concept of interest

The term “concept related” refers to ideas that share some connections with the concept at hand but do not fully embody it. Yet, the notion in question interacts with similar concepts [12]. The concepts of caring, caregiver, and burden commonly relate to family caregiving in the nursing and healthcare literature. For instance, nursing studies place a lot of emphasis on the theory of caring. Some nursing scholars have argued that caring distinguishes nursing from other health care professions [68]. Understanding, being with, acting for, enabling, and sustaining faith in the patient's ability to attain health-related goals are all parts of Swanson's caring processes, an approach to nursing practice. So, in this context, caring is more closely associated with the professional acts of healthcare providers taken towards patients and their families. Another idea that is frequently seen and used while discussing caring is caregiver and burden. Whereas the terms have various facets. Caregiver, used as a noun in this context, is the actor, while caregiving, is utilized as a verb. For more details, see Table .3 below.

**Table 3:** The definition of a related concept.

Related Concept	Definition	Source
*Caring*	Central to human expertise, to curing, and healing was extended	(*The Primacy of Caring: Stress and Coping in Health and Illness* (Benner & Wrubel, 1989)
	It encompasses tending, playing, and learning, which can build trust, address patient needs, promote physical and spiritual well-being, and foster a sense of being in growth to assist the body's healing processes.	(Eriksson, 1997)
*Caregiver*	The legal definition of a primary caregiver is someone older than 18 years old and responsible for providing direct care or assistance to someone unable to perform critical tasks necessary for everyday survival.	(US Legal Law)
	“A person who provides direct care (as for children, elderly people, or the chronically ill)”.	*Merriam Webster's Dictionary*
*Burden*	It is defined into subjective and objective burdens. While the objective burden is defined as occurrences or actions connected to poor caring experiences, the subjective burden is primarily concerned with the personal feelings of caregivers generated while executing the caring function	*(Hoenig & Hamilton, 1966)*
	“The extent to which caregivers perceived their emotional, physical health, social life, and financial status as a result of caring for their relative”p. 261	(Zarit et al., 1980)

### 3.6 Identify a model case

Choosing a model case gives the researcher a precise illustration, enabling the researcher to develop a more concrete understanding of the idea and defining the course of the research (Rodgers BL, Knafl KA.2000). The following is a case example of family caregiving.

Mr FR, a Muslim, is the first child of Mr PT, who has had Parkinson's disease with dementia for the past seven years. In line with Mr PT's motoric and cognitive decline, Mr FR received a scholarship this year to further his education. So he has now decided to forego pursuing a Master's degree. He understood that, following his religious teaching, he was responsible for caring for his father. He acknowledged that since fulfilling the family caregiving role, he had to make numerous sacrifices, but he persisted despite the first shock, feeling unprepared, frustrated, and experiencing other drawbacks. He claims to have paid attention and surrendered to Allah's will.

He was the one who assisted his father financially, physically, and his younger siblings and uncle also provided support. Together, they promised to keep their father out of nursing homes. He put his faith in Allah's power. So, despite the most significant challenges, he has continued to engage in acts of worship and prayer. Because this was a gift from his Lord, he had faith that Allah would support him through it all.

Mr FR is aware of the many blessings that had come since he had done his role. Starting with the chance to work somewhere that pays well, even though it is not his ideal position, then finding a wife who is sympathetic to his predicament and wants to assist him in caring for his father. Also, he now enjoys his voluntary work as a speaker at numerous seminars for Parkinson's disease family caregivers. He was deeply convinced that what he was doing demonstrated his allegiance to his Lord, and that Allah would reward him here and in the hereafter.

## 4 Discussion

In the Qur'an and Hadith there is no precise definition of family caregiving, even though the Islam religion has a very strong emphasis on piety [69]. Suggestions related to family caregiving are included in Ri'ayah or in other words “safeguarding”, where humans are revered as maintainers of themselves, others, and the universe as a whole. As in the Hadith from a quote by Abdullah ibn Umar: ‘‘The Messenger of Allah, peace and blessings be upon him, said, ‘Every one of you is a shepherd and is responsible for his flock. The leader of people is a guardian and is responsible for his subjects. A man is the guardian of his family, and he is responsible for them. A woman is the guardian of her husband's home and his children, and she is responsible for them. The servant of a man is a guardian of the property of his master, and he is responsible for it. No doubt, every one of you is a shepherd and is responsible for his flock” [[54], [55], [56]].

Hence, it is clearly stated that guarding is a responsibility attached to every Muslim who is a believer. This is in line with the results of the analysis of the articles that indicated family caregiving is considered a heavy duty and a religious responsibility [31,60]. The duties performed by the family caregiver also maintain the faith of the caretaker by making sure the caretaker continues to carry out their worship [25]. In line with this study, participants from various cultures and religions in Canada argued that spiritual beliefs were deeply ingrained in their lives and were clearly a part of how they defined themselves and their sources of hope and comfort. Their faith was visible in their practises (such as prayer and scripture reading) and influenced how they coped with advanced illness [70]. Their faith clearly provided them with the strength to deal with what was happening to them [71].

Cultural and religious beliefs, particularly filial piety, can significantly influence the definition of family caring. Similar to the teachings of Confucius, which became established throughout Asia. The scholars also attempted to investigate and juxtapose the Confucian notion of filial devotion with the teachings of Islam and Christianity [69]. Several motivations have been identified in the literature that led children to care for their elderly parents in a variety of settings [59,[72], [73], [74]]. Respect, a desire to maintain family harmony, love and affection for parents, a sense of responsibility on the part of the children, and a desire to repay their parents for their physical and financial sacrifices, are among these [74]. Apart from the statement above, moral and humanitarian goals are also things that deserve to be included, as thirteen articles mentioned the involvement of the extended family and community in family caregiving. This happened because in the Qur'an Ali Imran: 103, Hud:46 it is stated that fellow Muslims are brothers, brothers in faith [53]. Furthermore, the size of the family caregiving network was arguably one of the factors that influenced caregiver distress. Caregivers who were assisted in caring for an elderly parent by other family members reported a lower caregiving burden [75,76]. However, interestingly, in Islamic sources [77,78], it was stated that family caregiving especially with the elderly and people with disabilities, is becoming one of the government's responsibilities.

Although having a devastated life seems inevitable in family caregiving experiences, all articles mentioned that caregivers keep doing it, in spite of this. This action is assumed to be related to their belief in rewards as mentioned in the Al Quran “Indeed, Allah does not do injustice, [even] as much as an atom's weight; while if there is a good deed, He multiplies it and gives from Himself a great reward” (53). This enables the family able to move on and live with hope. Viewed from another angle, optimism has been shown to be associated with maintaining quality of life [79]. Furthermore, it is claimed that optimism is also important for patients to establish a realistic expectation and spend their remaining time meaningfully [80].

Examining the above concept of family caregiving from an Islamic perspective shows that these theories are based on humanism and the notions of family caregiving is based on a humanistic conceptualization. The humanistic nature of Islam, as reflected in the results, suggests that Islam is preoccupied with maintaining a balance between faith, worship and morality. These three components must be in harmony to create harmony in life [8,54,64,81]. Providing sufficient support to patients and their family caregivers is considered important to uplift the burden of caregivers and make the patients' disease outcomes better. In the same vein, Buddhists perceive care as a fundamental human endeavour, encompassing affection, reliance, dedication, and the cultivation of positive connections. Their priorities include the significance of a hereafter, the state of one's spiritual health, and the protection of an individual's autonomy [82].

The fact that related terms for family caregiving shared characteristics with caring or caregiving, looking after, shows multidimensionality, non-specificity, and cumulative impacts, and demonstrates their close association. Utilizing a small set of operational caring criteria specific to the family scope concept, to differentiate between these terms is insufficient to establish that family caregiving is a distinct concept. Hence, we underline the need for additional empirical research on the characteristics and determinants of family caring, particularly within the Islamic religious community. Our research advances the understanding of family caring as a concept, despite the fact that we only included documents published in English for this analysis and only from both Arabic and English Islamic sources. Documents in other languages, as well as unpublished data or teaching resources on family caregiving were not included in the analysis.

We believe it is crucial to determine the appropriate concept of family caregiving for the those in Muslim communities who are faithful to the Islamic religion. This is because of the rising significance of family involvement in home-based health care and the possibility of recurrence of pandemic diseases such Covid-19 which has been experienced recently. We believe this research gives a thorough definition and comprehensive evidence of the Islamic (religious) notion of family caregiving for the Muslim communities in different countries. In light of the beneficial consequence and antecedent of family caregiving, policymakers might use the findings of this study to strengthen their support systems. Training or support programmes for family caregivers to acquire knowledge and abilities in home care for family members are an important component of this. Knowledge, abilities, and a feeling of support are crucial to the long-term success of home care. Hence, family caregivers can continue to provide care while preserving their wellbeing. In addition, the findings of this study can inform the development of family nursing education curricula, as well as guidelines and evaluation tools for families caregivers in the various Muslim communities who wish to be are faithful to the Islamic religion.

## 5 Conclusion

In Islamic and Muslim communities, the concept of family caregiving is considered significant since it is regarded as a gift, a religious responsibility, and a commitment that lasts a lifetime. In the comfort of their own homes, Muslim family caregivers offer comprehensive care for their family members. This care includes nursing care, care for the body, care for the mind, and care for the spirit. The moral and human reasons of extended families or communities as family carers are the most essential features in this study. Additionally, it is necessary to obey Allah and believe in the ideas of Islamic fate. It is also a significant aspect of this study. The information that this study provides contributes to a new discourse on family caregiving that is based on Islamic literature. Additionally, it helps facilitate comprehension of the practices that are followed by Muslim communities all over the world.

## Funding

This research did not receive any specific grand from funding agencies in the public, commercial, or not – for profit sector.

## Author contributions

Martyarini Budi Setyawati: Conceptualization, Data curation, Formal analysis, Investigation, Methodology, Project administration, Writing – original draft. John Parsons: Conceptualization, Methodology, Supervision, Writing – review & editing. Bobbi Laing: Data curation, Formal analysis, Supervision, Writing – review & editing. Andrew Lynch: Data curation, Supervision, Writing – review & editing. Imam Labib Hibaurrohman: Formal analysis, Investigation. Farah Nurril Izza: Conceptualization, Data curation, Formal analysis, Writing – review & editing

## Data availability statement

This concept of analysis provides a detailed examination of the data that already exists in a database.

## Declaration of competing interest

The authors declare that they have no known competing financial interests or personal relationships that could have appeared to influence the work reported in this paper.
