# Supplementary material for: The family caregiving; A Rogerian concept analysis of Muslim perspective & Islamic sources
Source: Heliyon. 2024 Jan 28;10(3):e25415. doi: 10.1016/j.heliyon.2024.e25415 (PMC10865263; doi:10.1016/j.heliyon.2024.e25415)
Supplement: Multimedia component 1 [file mmc1.docx]

**Title:** **The Family Caregiving; A Rogerian Concept Analysis of Muslim Perspective & Islamic Sources**

**Electronic searches:** CINAHL, PubMed, Ovid Medline, Scopus

**SPIDER criteria**

Inclution Ctiteria

| (S) Sample | Family who look after their sick family vulnerable family member.  Looking after here is provide assistance related to an underlying [physical](https://en.wikipedia.org/wiki/Physical_disability) or [mental disability](https://en.wikipedia.org/wiki/Disabilities_affecting_intellectual_abilities) for at-home care delivery and assist in the activities of daily living (ADLs) who are unpaid  Key term :  “famil*” OR “relative*” OR “parent*” OR “sibling*” OR “spouse*” OR “kinship*” OR “husband*” OR “wife*” OR “sister*” OR “brother*” |
| --- | --- |
| (PI) Phenomenon of Interest | Caregiving on Muslim family  .Key term :  Caregiving OR caring OR “care*” OR “treat*” OR “tak* care*” OR look after’ AND “Islam*” OR “Muslim*” OR Oman OR Qatar OR Kuwait OR Iran OR Iraq OR UEA OR Saudi Arabia OR Saudi OR Indonesia OR Pakistan |
| (D) Study Design : | Data were collected using open questionnaires, surveys, interviews, focus groups or case studies.  Key term :  “questionnaire*” OR “interview*” OR “focus group*” OR “case stud*” |
| (E) Evaluation : | Only those that present perceptions, attitudes, experiences, values, or concepts.  Key term :  “view*” OR “experienc*” OR “opinion*” OR “attitude*” OR “perce*” OR “belie*” OR “concept*” |
| (R) Research type | Only peer-reviewed journal articles written in English were included. |

**Exclusion criteria:**

- Studies were excluded if the caregivers were professionals.
- It only shows the provision of parenting/educational setting
- In a hospital or hospice setting
- The care recipient dying or already gone
