# Supplementary material for: The family caregiving; A Rogerian concept analysis of Muslim perspective & Islamic sources
Source: Heliyon. 2024 Jan 28;10(3):e25415. doi: 10.1016/j.heliyon.2024.e25415 (PMC10865263; doi:10.1016/j.heliyon.2024.e25415)
Supplement: Multimedia component 2 [file mmc2.docx]

**SEARCH STRING**

| **Database** | **Date of search** | **results** | **Search string** |
| --- | --- | --- | --- |
| CINAHL | 13/12/22 | 1640 | “view*” OR “experienc*” OR “opinion*” OR “attitude*” OR “perce*” OR “belie*” OR “concept*”  AND  “famil*” OR “relative*” OR “parent*” OR “sibling*” OR “spouse*” OR “kinship*” OR “husband*” OR “wife*” OR “sister*” OR “brother*” – abstract  AND  caring OR “care*” OR “treat*” OR “taking care*” OR look after’ – abstract  AND  “Islam*” OR “muslim*” OR Oman OR Qatar OR Kuwait OR Iran OR Iraq OR UEA OR Saudi Arabia OR Saudi OR Indonesia OR Pakistan  AND – abstract  “questionnaire*” OR “survey*” OR “interview*” OR “focus group*” OR “case stud*” |
| Pubmed | 13/12/22 | 922 | (("famil*"[Title] OR "relative*"[Title] OR "parent*"[Title] OR "sibling*"[Title] OR "spouse*"[Title] OR "kinship*"[Title] OR "husband*"[Title] OR "wife*"[Title] OR "sister*"[Title] OR "brother*"[Title]) AND (caring[Title/Abstract] OR "care*"[Title/Abstract] OR "treat*"[Title/Abstract] OR "taking care*"[Title/Abstract] OR look after’[Title/Abstract])) AND ("Islam*"[Title/Abstract] OR "muslim*"[Title/Abstract] OR Oman[Title/Abstract] OR Qatar[Title/Abstract] OR Kuwait[Title/Abstract] OR Iran[Title/Abstract] OR Iraq[Title/Abstract] OR UEA[Title/Abstract] OR Saudi Arabia[Title/Abstract] OR Saudi[Title/Abstract] OR Indonesia[Title/Abstract] OR Pakistan[Title/Abstract]) |
| Ovid Medline | 12/12/22 | 786 | ((caring OR care* OR treat OR take* care OR look* after[mp= abstract]) AND (“Islam*” OR “muslim*” OR Oman OR Qatar OR Kuwait OR Iran OR Iraq OR UEA OR Saudi Arabia OR Saudi OR Indonesia OR Pakistan) AND (family OR families OR relatives OR parents OR siblings OR caregiver OR spouse* OR kinship OR husband OR wife [mp=title])). |
| Scopus | 12/12/22 | 360 | ( TITLE-ABS-KEY ( famil* OR relative* OR parent* OR sibling* OR spouse* OR kinship* OR husband* OR wife* OR sister* OR brother* ) AND TITLE-ABS-KEY ( caring OR care* OR treat* OR taking AND care* OR look AND after ) AND TITLE-ABS-KEY ( islam* OR muslim* OR oman OR qatar OR kuwait OR iran OR iraq OR uea OR saudi AND arabia OR saudi OR indonesia OR pakistan ) ) |
|  |  | 3708 |  |
